# Implementing Symptom Management Follow-up Using an Electronic Patient-Reported Outcome Platform in Outpatients With Advanced Cancer: Longitudinal Single-Center Prospective Study

**DOI:** 10.2196/21458

**Published:** 2022-05-10

**Authors:** Lili Tang, Yi He, Ying Pang, Zhongge Su, Jinjiang Li, Yening Zhang, Xu Wang, Xinkun Han, Yan Wang, Zimeng Li, Shuangzhi He, Lili Song, Yuhe Zhou, Bingmei Wang, Xiumin Li

**Affiliations:** 1 Key Laboratory of Carcinogenesis and Translational Research (Ministry of Education) Department of Psycho-oncology Peking University Cancer Hospital & Institute Beijing China; 2 ePRO Vision Health Technology Co, Ltd Beijing China

**Keywords:** electronic patient-reported outcome, symptom management, advanced cancer, outpatient, follow-up

## Abstract

**Background:**

Patients with cancer experience multiple symptoms related to cancer, cancer treatment, and the procedures involved in cancer care; however, many patients with pain, depression, and fatigue, especially those outside the hospital, receive inadequate treatment for their symptoms. Using an electronic patient-reported outcome (ePRO) platform to conduct symptom management follow-up in outpatients with advanced cancer could be a novel and potentially effective approach. However, empirical evidence describing in detail the preparation and implementation courses in a real setting is needed.

**Objective:**

The purpose of this paper was to describe the implementation process and evaluation of an ePRO platform that facilitates symptom management for patients with cancer, share our experiences and the problems we encountered during the process of implementation, and share the solutions we identified for those problems. Moreover, we tested the feasibility, safety, and efficacy of the ePRO platform.

**Methods:**

This was a real-world, ongoing, longitudinal, single-center, prospective study with a total of 7 follow-ups conducted within 4 weeks after the first visit to the symptom management clinic (on days 1, 3, 7, 10, 14, 21, and 28). Participants were encouraged to complete scales for physical symptoms (pain, fatigue, and shortness of breath), cognitive symptoms (memory problems and impaired concentration), and affective symptoms (especially depression and anxiety) during follow-up. The design and function of the ePRO-doctor client and ePRO-patient client, the patient-reported outcome (PRO) scales used in the study, and the strategies to promote symptom tracking have been described. Moreover, the training and evaluation for research assistants have been presented. The efficacy of the ePRO platform was assessed with a comparison of the baseline and 4-week outcomes on the MD Anderson Symptom Inventory.

**Results:**

Using the ePRO platform for symptom management follow-ups in advanced cancer patients was associated with a high completion rate (72.7%-86.4%) and a low drop-off rate (23.6%). The ePRO platform sent 293 alert notifications to both patients and doctors, which promoted patient security. The short and sharp PRO tool selection, user-friendly interface, automatic reminder notifications and alerts, and multiple dimensional training were essential components for the preparation and implementation of the ePRO system. The results showed significant improvements in the mean scores of pain, fatigue, and numbness from baseline to day 28 (*P*=.02, *P*=.02, and *P*<.001, respectively).

**Conclusions:**

The use of an ePRO platform for symptom management follow-ups in advanced cancer patients is time-saving, energy-saving, and effective. PRO tool selection, platform design, and training of research assistants are important aspects for implementation. Future research should validate the ePRO platform in a larger randomized controlled study.

## Introduction

Patients with advanced or metastatic cancer usually have severe symptom burden, which is significantly higher than that in patients with no evidence of cancer metastasis [1]. Symptom burden has also been found to be correlated with treatment-related factors. About one-third of advanced cancer patients were found to have persistent severe symptom burden during chemotherapy [2], and high symptom burden was found to be negatively associated with patients’ psychological status, function, and quality of life [3,4].

However, research on symptom management has mainly focused on inpatients. Relevant research for outpatient symptom management has been limited. Traditional outpatient follow-up is usually via email or telephone, but a low response rate is a common problem in these 2 modes.

In China, since the average length of hospitalization has shortened dramatically, especially in some top cancer centers, much works on symptom management has been carried out in the outpatient department [5]. Symptom management in outpatients has some difficulties. First, outpatients only come to the clinic at a certain time point. Most of the time, they are outside of the hospital, and there is a lack of monitoring of their situations. Second, the means of communication between outpatients and doctors are limited. Many patients only come back to see their doctors when their symptoms become very serious. In some cases, patients cannot get timely and effective symptom management due to various factors, even though their symptoms are very serious. The poor situation of symptom management creates a burden for not only patients but also their families and caregivers, and it even introduces huge burdens of medical resources and costs. Unmet care needs may also decrease patient adherence to treatments [6]. A recent study [7] showed that a web-based app can improve symptom management and adherence for aromatase inhibitors in breast cancer patients.

Patient-reported outcomes (PROs) assess the problems a patient can report about his or her own experiences. These include symptoms, functioning, and mental health. However, a key barrier of using PRO data in clinical settings is the limitation of paper-based questionnaires, which cannot be transformed into instantly accessible information. Compared with traditional paper and pen testing, the electronic patient-reported outcome (ePRO) platform has the advantages of data collection standardization and quality management [8]. An effective ePRO platform can monitor the symptoms of patients outside the hospital better, give a timely alarm, and facilitate timely symptom management; therefore, the ePRO system can improve symptom management in outpatients. In recent years, many techniques of ePRO system design have been greatly developed, such as data transmission, storage, confidentiality, applicability, and convenience. Traditional electronic platforms are mainly based on an email system, while the new generation of ePRO platforms is mainly based on smartphones [9,10].

Most ePRO systems are treatment-centered and have been designed to serve a special kind of treatment [8,11,12]. In the selection of PRO tools, most involve treatment-related symptoms, and follow-up frequency and interval are set for the treatment. So far, an ePRO-based symptom management follow-up system for patients with advanced cancer generally is lacking. Nowadays, in China, the access rates of the internet and smartphones are very high. In 2018, an ePRO symptom management research project was launched in Peking University Cancer Hospital, which included a single-institute longitudinal study and a multi-center cross-sectional study. In the longitudinal study, we aimed to monitor the symptoms of outpatients with advanced cancer using an ePRO symptom management follow-up system based on a smartphone. The purpose of this paper was to describe the implementation process and results, present the advantages of the ePRO system, and share our experiences and the problems we encountered during the process of implementation, as well as the solutions we identified to solve the problems.

## Methods

### ePROhub, ePRO-Doctor Client, and ePRO-Patient Client

#### ePROhub

ePROhub provides the primary function of collecting data through PRO tools, as well as adapting and managing the data. Because of the connection with the hospital information system, the ePRO data are more convenient to be managed and analyzed together with other medical data. Intelligent operations include generating and managing the accounts of doctors and patients, collecting and checking patients’ information, sending follow-up reminders, and alerting automatically about serious symptom. This platform also has an electronic signature system that could be used for both subjects (sign informed consent) and research assistants (sign after each subject’s enrollment and follow-up to improve research quality management).

#### ePRO-Doctor Client

**Figure 1:**
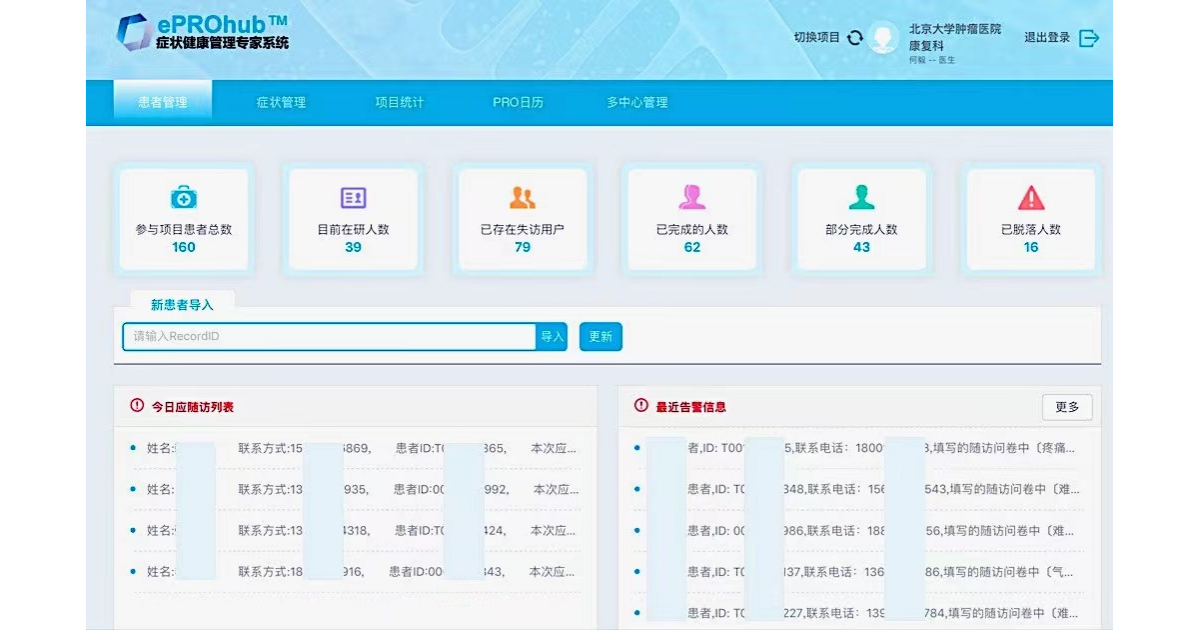
Doctor’s interface for research management.

The doctor-client interface shows the important information of the program, such as the number of participants, the number of participants completed, the number of participants partly completed, and the number of drop-offs. The interface is updated in real-time to present the latest progress of the program for doctors and researchers. There is a list of people who need to be reminded to do the follow-up survey and another list of people who have symptoms over the alert level. In this study, according to the cut-off point of the MD Anderson Symptom Inventory (MDASI; ≥7), the platform sent an alarm to the ePRO-doctor client. Each doctor or researcher could view his/her own participants, and the management staff could view the entire enrollment situation (Figure 1).

#### ePRO-Patient Client

**Figure 2:**
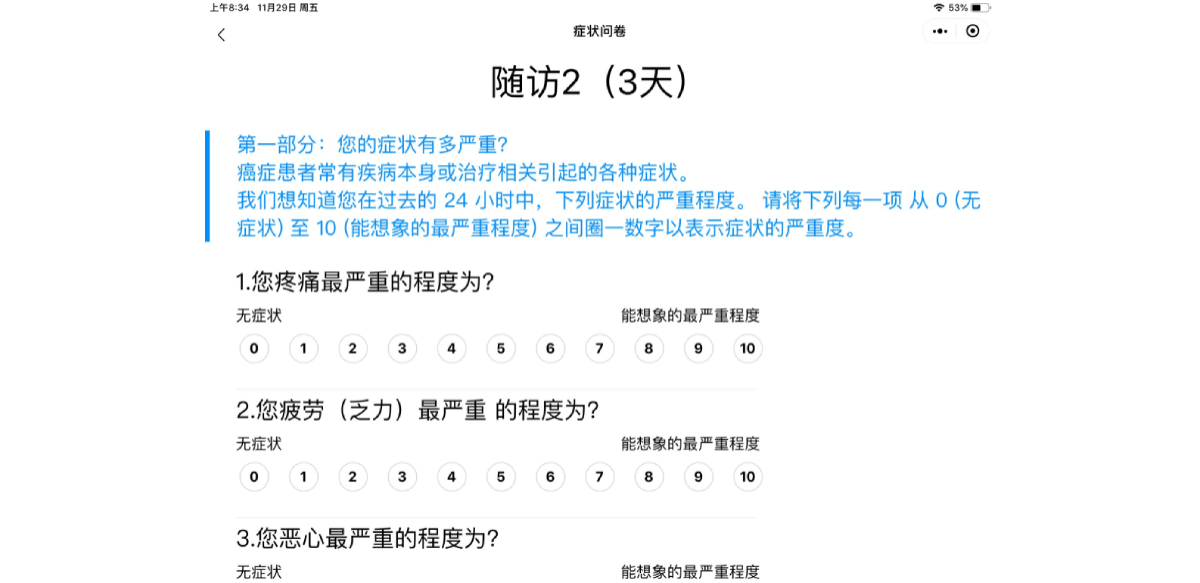
Patient's interface of patient-reported outcome reporting.

The interface has been designed as a touch screen, which conforms to patients’ usage habits. Due to the limitation of the screen size of a mobile phone, the PRO scale has been designed to display in the horizontal direction, which is in line with the users’ experience to the maximum extent. There are only 1 or 2 questions on a page, making it easier to read, and the page could be enlarged, making it easier for patients to touch the screen to choose their answers (Figure 2). PRO data could be reported by patients anywhere through an applet based on the WeChat app, which is the most popular social app, without restrictions on the type of smartphone operating system. The ePRO system could identify how many times patients had fulfilled and matched the right scales. All the data were uploaded to a database established in Peking University Cancer Hospital. A strict encryption system was used to ensure data security.

### PRO Scales

We used several validated instruments in the multi-dimensional ePRO system, which are presented below.

#### MDASI

The MDASI [13,14] is a widely used symptom inventory with 19 items (13 items for symptom severity and 6 items for life interference; 0=nothing to 10=most severe). A psychometric study has revealed that the Chinese version of the MDASI has good reliability and validity. Moreover, we added 5 more items for specific cancer sites in our study to capture the special characteristics (constipation was added for all cancers, hot flash and upper limb lymphedema were specific for breast cancer, cough was specific for lung cancer, and swallowing difficulty was specific for esophagus cancer). Compared to the European Organization for Research and Treatment of Cancer Quality of Life Questionnaire (EORTC-QLQ) [15], which needs at least 1 week between 2 follow-ups, we used the MDASI as our screening tool, which could be used every day, because we needed to monitor symptoms at a high frequency.

#### Insomnia Severity Index

There are a total of 7 items in the Insomnia Severity Index (ISI; 0-4 score for each item, with a sum score of 28). The ISI is a validated scale for measuring insomnia severity in the last 2 weeks. Scores of 0-7 indicate no insomnia, 8-14 indicate subclinical insomnia, 15-21 indicate moderate insomnia, and 22-28 indicate severe insomnia. The simplified Chinese version of the ISI has been validated by Lin et al [16].

#### Hospital Anxiety and Depression Scale

The Hospital Anxiety and Depression Scale (HADS) has 14 items with a score spectrum of 0-4 for each item, which is used to measure the anxiety and depression of patients in the past week. It is more commonly used for patients with somatic symptoms in general hospitals, with good reliability and validity, and is recommended for use in patients with advanced cancer or those receiving palliative care [17].

#### Patient Health Questionnaire-9 Items

The Patient Health Questionnaire-9 Items (PHQ-9) is used to evaluate depression in patients in the past 2 weeks. The score spectrum of symptom severity is from 0 (none at all) to 3 (almost every day), and the total score is from 0 to 27. Depression can be considered when the sum score is ≥10. The simplified Chinese version of the PHQ-9 has good validation [18].

#### EuroQol 5 Dimensions Questionnaire-5L Version

The EuroQol 5 Dimensions Questionnaire-5L Version (EQ-5D-5L) is a multidimensional measurement for health-related quality of life, which contains the following 5 domains to describe patients’ health: (1) mobility, (2) self-care, (3) usual activities, (4) pain/discomfort, and (5) anxiety/depression, with a scale from 0 (no difficulty) to 4 (extreme difficulty) [19,20]. The Functional Assessment of Cancer Therapy: General (FACT-G) [21] contains too many items, and some of them are easily avoided by patients. We used EQ-5D-5L to measure the quality of life because it has fewer items and has convenient access to get reliable results.

#### Distress Thermometer

Distress Thermometer is recommended by the National Comprehensive Cancer Network in the distress management guideline. It has only 1 item with a scale from 0 (no distress) to 10 (extreme distress). The problem list includes the following 5 domains: practical problem, communication problem, emotion problem, physical problem, and spirit and religion problem. It is recognized as the briefest tool for distress screening, especially in busy oncology clinical practice [22].

### Symptom Tracking Promotion Strategies

Enrolled patients completed the baseline assessments and accepted ePRO standard operating procedure training when they first visited the symptom management clinic. Baseline assessments included demographic and medical information, symptom situation, and medication situation. The ePRO system application training included instructions on how to log into the ePRO system, and how to report their symptoms and other medication situations. In order to improve follow-up compliance, the system sent a message automatically to remind patients at 8 AM on each follow-up day (1st, 3rd, 7th, 10th, 14th, 21st, and 28th day after the first clinic visit). If the follow-up self-report was not completed at 4 PM, the system automatically sent a message again. If there was still no response, research assistants would call them again the next day and record the reasons for noncompletion. If the patient was not connected after 2 calls, the data of this follow-up session were regarded as “lost.”

A time window of 24 hours before and after each follow-up was set, and the system recorded all the completion time points of the patients. Additionally, the system set the alert function. If the score of symptoms reported by the patients exceeded the cutoff value, the system reminded the patients to see a doctor in time.

### Training and Evaluation for Research Assistants

Making a standardized operation process manual and an operation video for research assistants is convenient for them to study. The group training was organized for one time, while individual (one-to-one) training was carried out one-to-one. After the training, all research assistants were required to pass a test of practical operation to get started on their official work.

There was a question and answer session to solve operation problems after enrollment of around 10 cases. In addition, the practical problems faced by research assistants were shared in the WeChat working group at any time.

Before application in clinical practice, some evaluations were carried out, including running the trial test for the ePRO system and its supporting system, testing the function of SMS text message notifications for patients, confirming the process of patients’ online follow-up, updating the layout of the doctor version of the ePRO system, and switching the testing system database to the formal project database.

### Study Population, Eligibility Criteria, and Recruitment

All eligible patients who visited the symptom management clinic for the first time were invited to participate in the study by the doctors in the clinic. The inclusion criteria were as follows: (1) age over 18 years; (2) fluency in Chinese; and (3) confirmed diagnosis of advanced lung cancer, liver cancer, gastric cancer, esophageal cancer, colorectal cancer, or breast cancer. The exclusion criteria were as follows: (1) a history of major severe mental disorders (unable to cooperate with the investigator); (2) being in poor physical condition, as judged by the attending physician (not able to complete the whole study); and (3) being unable to use the ePRO platform.

### Data Collection and Process

**Figure 3:**
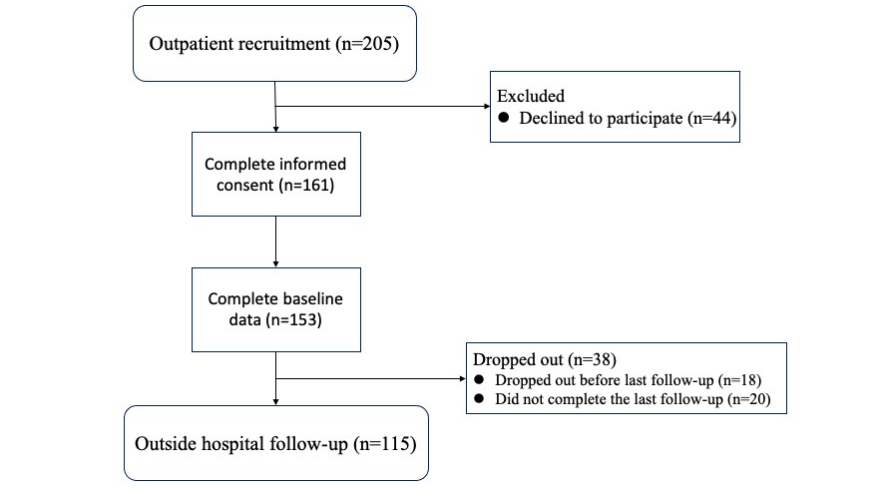
Study flowchart.

Completed data with both PROs and other information were collected at baseline (day 0, patient’s initial visit) and follow-ups conducted within 4 weeks after the first visit to the symptom management clinic (days 1, 3, 7, 10, 14, 21, and 28), using an ePRO platform system supported by the research team and ePRO Vision. The flow chart of the study is shown in Figure 3.

PRO data were collected using an ePRO platform. The system could recognize the individual scores of MDASI items due to the cutoff points that we set up. For those scales that needed results to be calculated, such as PHQ-9, data were first captured by WeChat, saved in REDCap, and then calculated. The output data could be transferred to professional statistics programs, like SAS or SPSS, with a standard data format for the final data analysis. All data were deidentified and stored on the REDCap platform.

### Evaluation

The completion rate was defined as the proportion of patients who completed the self-report using the ePRO system within the stipulated time. The drop-off rate was defined as the proportion of patients who refused to complete the self-report using the ePRO system or failed to complete the last follow-up at day 28.

### Ethics and Consent

The original study was approved by the Institutional Review Board of Peking University Cancer Hospital on February 13, 2019 (study #2019YJZ07). All participants provided written informed consent.

### Statistical Analysis

Baseline characteristics were summarized using mean and SD for continuous variables or number and percentage for categorical variables. Assessments for symptom (MDASI) characteristics were conducted for all patients, and a paired *t* test was used to determine whether there was a statistically significant change between the baseline and day 28 scores. SPSS software v26 (IBM Corp) was used to analyze the data. All *P* values were 2-sided, and *P*<.05 was considered statistically significant.

## Results

### Recruitment

Among 205 eligible patients with advanced cancer who were approached, 161 agreed to participate in the study and 153 completed the baseline assessment. Eligible patients refused to participate for various reasons, and the main reason was “I don’t want to be disturbed and it’s useless to improve my symptoms.” Patient characteristics are detailed in Table 1.

The completion rates were from 72.7% to 86.4% at each follow-up, and the highest completion rate was at follow-up 1 (day 1), while the lowest rate was at follow-up 6 (day 21) (Table 2).

**Table 1:** Disease and demographic characteristics of the participants (N=153).

Variable		Value
Age (years), mean (SD)		56.3 (11.0)
Age range (years)		27-86
**Gender, n (%)**		
	Male	85 (55.6)
	Female	68 (44.4)
**Ethnic group, n (%)**		
	Han	139 (90.8)
	Other	14 (9.2)
**Education, n (%)**		
	Junior high school or below	48 (31.4)
	High school/secondary school	44 (28.8)
	Undergraduate/college	56 (36.6)
	Master’s degree or above	5 (3.3)
**Cancer diagnosis, n (%)**		
	Breast	16 (10.5)
	Gastric	20 (13.1)
	Esophagus	10 (6.5)
	Liver	12 (7.8)
	Lung	48 (31.4)
	Colorectal	47 (30.7)
**Disease status, n (%)**		
	Progressive	91 (59.5)
	Partial response	8 (5.2)
	Stable	38 (24.8)
	Unclear	16 (10.5)
**Disease stage, n (%)**		
	Metastatic	142 (92.8)
	Locoregional	11 (7.2)
**Eastern Cooperative Oncology Group score, n (%)**		
	0	39 (25.5)
	1	71 (46.4)
	2	28 (18.3)
	3	15 (9.8)
**Current anticancer therapy, n (%)**		
	No	61 (39.9)
	Yes	90 (58.8)

**Table 2:** Completion rate and missing rate at each follow-up (N=153).

Time point	Completion, n (%)	Missing, n (%)
Baseline (day 0)	153 (100%)	0 (0%)
Follow-up 1 (day 1)	132 (86.3%)	21 (13.7%)
Follow-up 2 (day 3)	128 (83.7%)	25 (16.3%)
Follow-up 3 (day 7)	125 (81.7%)	28 (18.3%)
Follow-up 4 (day 10)	122 (79.7%)	31 (20.3%)
Follow-up 5 (day 14)	123 (80.4%)	30 (19.6%)
Follow-up 6 (day 21)	111 (72.5%)	42 (27.5%)
Follow-up 7 (day 28)	120 (78.4%)	33 (21.6%)

### Feasibility

Overall, 43.5% (263/604) person-time follow-up assessments were completed by patients automatically before the notification was sent by the ePRO system, 42.6% (257/604) were completed within 8 hours after the first reminder message was sent, and 13.9% (84/604) were completed after the reminder phone call of a research assistant.

The drop-off rate was 23.6% (38/161) in the longitudinal study. Eighteen patients dropped off before the last follow-up (day 28), while 20 patients did not complete the last follow-up assessment. Among them, 14 patients rejected participation in the follow-ups continually, 19 patients could not be contacted, 3 patients died, 1 patient was considered not capable of participating in this study continually by a doctor, and 1 patient dropped off for an unknown reason.

The ePRO system sent a total of 293 alert notifications to both doctors and patients when the patient-reported symptom severity reached the altered score.

In the cohort, 153 patients underwent symptom assessments with the MDASI, HADS, ISI, and PHQ-9 scales at baseline. Of these 153 patients, 119 (77.8%) underwent reassessment at day 28, and we observed significant decreases in the mean scores of pain, fatigue, and numbness from baseline to day 28 (*P*=.02, *P*=.02, and *P<*.001, respectively) (Table 3).

**Table 3:** Symptom assessment scores between baseline and day 28.

Assessment	Baseline score, mean (SD)	Day 28 score, mean (SD)	*P* value
Pain	4.91 (3.3)	3.52 (2.7)	.02
Fatigue	5.12 (2.8)	4.29 (2.8)	.02
Nausea	2.52 (2.7)	2.29 (2.5)	.32
Disturbed sleep	5.32 (2.9)	3.94 (2.7)	.50
Distress	4.42 (3.0)	3.28 (2.8)	.31
Shortness of breath	3.14 (2.7)	2.70 (2.7)	.79
Difficulty remembering	3.30 (2.6)	2.65 (2.4)	.10
Lack of appetite	4.32 (2.9)	3.31 (2.9)	.89
Drowsiness	3.69 (2.8)	3.08 (2.7)	.38
Dry mouth	3.78 (2.8)	2.92 (2.6)	.19
Sadness	3.58 (3.1)	3.03 (2.9)	.24
Vomiting	2.03 (2.7)	1.64 (2.4)	.06
Numbness	3.74 (3.2)	3.14 (2.6)	<.001

## Discussion

### Advantages of Using an ePRO System

Using an ePRO system for symptom management follow-up had a lot of advantages. First, compared with the paper-pencil test, the ePRO reporting interface is much more friendly to seniors with poor sight, and the size of the font could be enlarged to make reading easier. Second, the system could remind patients that there are items missing answers automatically and could improve data integrity. Third, the system’s automatic reminder for each follow-up was very effective, and over 40% of patients completed the follow-up assessments within 8 hours after the first automatic reminder was sent, which improved the completion rate greatly and saved the time of the research assistants when compared with traditional follow-up by telephone, mail, or email [23].

Studies showed that the real-time symptom severity alarm function has an important role in symptom management [24,25]. During the study period, nearly 300 alter notifications were sent, so this function was very necessary for symptom management.

The ePRO system made research management easier. The researchers could see the real-time research progress of their clients. Researchers at different levels saw different kinds of reports, which not only made their research management easier but also protected the confidentiality of the research.

### Experiences of Implementation

#### Consideration for ePRO Platform Design

##### Follow-up Times

Follow-up times should be determined according to the study purpose. Our platform was designed to complete high-frequency self-reports in a short period of time, with 7 follow-ups over 4 weeks. Several platforms have already been set up with their own follow-up time according to different aims such as a platform for posttreatment surveillance in head and neck cancer [26].

##### Flexibility

The electronic symptom management system applet was based on the most popular social app in China, WeChat, which can be used in any place with wireless internet or mobile network coverage, without the issue of different smartphone operating systems. This saves the cost of developing a new app and saves users the hassle of downloading one more app, and the app is convenient and totally free [27].

##### Alerts

For patients and medical researchers, alert information about symptoms can be sent in real-time, and the system can display reminders directly on the screen when the doctor logs into the platform. By contrast, other foreign ePRO platforms [28] send the medical staff an email notification as a reminder, which may delay the information.

##### Applicability for Interfacing With Other Systems

There is good interoperability with the REDCap system. For instance, the software company ESD (Evaluation Software Development) has been developing the CHES Platform (Computer-Based Health Evaluation System) [29], which is a specialized software dedicated to the assessment, storage, and processing of ePRO data.

##### Education

Education for patients is beneficial for better symptom management, like cancer-related pain [30], so we have added educational material and doctor’s advice into the applet, as well as referral tips. This could help patients and caregivers to learn the skills of symptom management.

##### Integration Capacity

Most previous platforms can be divided into the following 2 categories: (1) treatment center, which is a platform designed according to a certain treatment, such as a PRO platform in chemotherapy [31], and (2) patient center, which is a PRO platform designed for a certain patient population. This system is integrated with treatment-related (such as symptom management) and population-related (such as outpatients) aspects.

### Experiences of Training Research Assistants

Multiple training methods were combined before the study implementation, including self-training, one-to-one training, and together with training, which makes the whole training process more time-saving and more effective. The training was not stopped after the evaluation, and the question and answer session at the beginning of the study was very helpful for research assistants to solve the problems they faced in practice.

### Patient Adherence and Benefit

Several studies on patients who were followed up outside the hospital found that the traditional follow-up compliance was less than 50% [32,33]. In our study, the overall response rate of patients reached at least 70% in each follow-up, and there was an average response rate of 80.3% for all 8 out-of-hospital follow-ups in 2 months. Even in app-based studies, there has been a problem of a high dropout rate, and in intervention research, the dropout rate usually reached 60% [34,35]. It was suggested that we should pay attention to this problem in future intervention research.

Several studies found that integration of ePROs into routine cancer care was associated with increased survival compared with usual care [36,37]. This was an observational cohort study (no intervention), and it was found that patients had benefits for several symptoms. Symptom monitoring via ePROs following treatment for cancer was associated with increased benefits among patients.

### Limitations

This study introduced the use of an ePRO platform. In the initial process, only patients who were referred to the symptom management clinic were enrolled in the study (not all patients treated in the oncology clinic). At the same time, only patients with advanced cancers at 6 sites were included. Our next goal is to integrate the platform with patient records, and future research should extend to all cancer patients.

Although the current ePRO platform had relatively high compliance, future studies should continue to explore ways to address dropout in populations at a high risk of dropout, especially in an intervention study.

In addition, the prospective nature of the study presented a limitation, and there was only 1 group of patients and no control group.

### Conclusion

The use of an ePRO platform for symptom management follow-ups in advanced cancer patients is time-saving, energy-saving, and effective, which can improve the completion rate and decrease the drop-off rate. PRO tool selection, platform design, and training of research assistants are important aspects that require attention.

## Abbreviations

ePRO: electronic patient-reported outcome
EQ-5D-5L: EuroQol 5 Dimensions Questionnaire-5L Version
HADS: Hospital Anxiety and Depression Scale
ISI: Insomnia Severity Index
MDASI: MD Anderson Symptom Inventory
PHQ-9: Patient Health Questionnaire-9 Items
PRO: patient-reported outcome
